# Splenectomy Associated Changes in IgM Memory B Cells in an Adult Spleen Registry Cohort

**DOI:** 10.1371/journal.pone.0023164

**Published:** 2011-08-04

**Authors:** Paul U. Cameron, Penelope Jones, Malgorzata Gorniak, Kate Dunster, Eldho Paul, Sharon Lewin, Ian Woolley, Denis Spelman

**Affiliations:** 1 Pathology Services, The Alfred Hospital, Melbourne, Victoria, Australia; 2 Infectious Diseases Unit, The Alfred Hospital, Melbourne, Victoria, Australia; 3 Department of Immunology, Monash University, Melbourne, Victoria, Australia; 4 Department of Medicine, Monash University, Melbourne, Victoria, Australia; 5 Department of Infectious Diseases, Monash Medical Centre, Clayton, Victoria, Australia; 6 Centre for Virology, Burnet Institute, Melbourne, Victoria, Australia; 7 Department of Epidemiology and Preventive Medicine, Monash University, Melbourne, Victoria, Australia; University of California Los Angeles, United States of America

## Abstract

Asplenic patients have a lifelong risk of overwhelming post-splenectomy infection and have been reported to have low numbers of peripheral blood IgM memory B cells. The clinical value of quantitation of memory B cells as an indicator of splenic abnormality or risk of infection has been unclear. To assess changes in B cell sub-populations after splenectomy we studied patients recruited to a spleen registry (n = 591). A subset of 209 adult asplenic or hyposplenic subjects, and normal controls (n = 140) were tested for IgM memory B cells. We also determined a) changes in IgM memory B cells with time after splenectomy using the cross-sectional data from patients on the registry and b) the kinetics of changes in haematological markers associated with splenectomy(n = 45). Total B cells in splenectomy patients did not differ from controls, but memory B cells, IgM memory B cells and switched B cells were significantly (p<0.001) reduced. The reduction was similar for different indications for splenectomy. Changes of asplenia in routine blood films including presence of Howell-Jolly bodies (HJB), occurred early (median 25 days) and splenectomy associated thrombocytosis and lymphocytosis peaked by 50 days. There was a more gradual decrease in IgM memory B cells reaching a stable level within 6 months after splenectomy. IgM memory B cells as proportion of B cells was the best discriminator between splenectomized patients and normal controls and at the optimal cut-off of 4.53, showed a true positive rate of 95% and false positive rate of 20%. In a survey of 152 registry patients stratified by IgM memory B cells around this cut-off there was no association with minor infections and no registry patients experienced OPSI during the study. Despite significant changes after splenectomy, conventional measures of IgM memory cells have limited clinical utility in this population.

## Introduction

The most clinically significant complication of splenectomy is overwhelming post-splenectomy infection (OPSI) which occurs in about 1 in 500 patients per annum and has a mortality of 50% [1]–[3]. The organisms causing OPSI are the encapsulated bacteria including *Streptococcus pneumoniae*, with others such as *Haemophilus influenzae*, and *Neisseria meningitidis* less common [1]. Although risk of OPSI has been thought to be highest in the first two years following splenectomy it may occur at any time [2], [4] and can occur even in those who have been managed with an appropriate immunization regimen. OPSI in the presence of adequate immunization may result from infection with serotypes not covered by the vaccine or because the vaccine failed to elicit an adequate immune response [4]. Prophylactic antibiotics have been shown in a randomized controlled trial to be of clear benefit in children with sickle cell anemia [5] and are advised for adults following splenectomy in particular during the first 2 years post-splenectomy [6]–[8]. Poor compliance is often a limiting factor in the continuous use of antibiotic prophylaxis [1], [9]–[12]. An assay that would better stratify risk of OPSI would provide a useful measure to determine those in whom prophylactic antibiotics could be discontinued without significant risk.

One proposed assay of splenic B lymphoid function measures circulating IgM memory B cells; a population of B cells in the peripheral blood that express the memory cells marker CD27, high surface IgM and low levels of IgD and which have been found to be low in young children and in asplenia [13]. These cells have been shown to correspond phenotypically and by gene expression array to the splenic marginal zone B cells [14] and have been shown, albeit in limited numbers of subjects, to be low in asplenic populations at risk for OPSI [13], [15]–[23] as well as in those with Common Variable Immuno Deficiency (CVID) or hypogammaglobulinaemia on immunoglobulin replacement [15], [16], [24] or in individuals with HIV-1 infection [25]–[28].

In animal models T independent antibody responses have been shown to be largely dependent on the marginal zone B cells and B1 cells and these responses to particulate antigens effect early bacterial clearance [29], [30]. In man such natural antibodies against pneumococcal polysaccharide produced as T independent responses are able to bind across serotypes [31]. Humans have been thought to differ from mice in the lack of B1 cells and in some characteristics of marginal zone B cells. Human marginal zone B cells circulate as IgM memory B cells in blood [14] while they are restricted to marginal zone in mice [32]. Although marginal zone B cells have been shown to contribute to the generation of T cell dependent antibody responses [33] there is little evidence of deficient vaccine responses in splenectomized subjects [17]. Nevertheless marginal zones in splenic lymphoid tissue have been proposed as the predominant source of the circulating IgM memory cells or marginal zone B cells that can provide early T cell independent responses during bacteremia [13].

Since marginal zone B cells have been postulated to play a role in innate B cell defences we sought to determine the potential clinical value of measurement of circulating IgM memory B cells after splenectomy as a biomarker of loss of splenic tissue by examining the distribution of memory B cell subsets in a cohort of splenectomized or hyposplenic subjects enrolled in a spleen registry. We compared the distribution of memory B cell subsets to healthy controls. We showed that the kinetics of loss of IgM memory B cells after splenectomy was delayed compared to the earlier onset of lymphocytosis, thrombocytosis and appearance of red cell changes including appearance of HJBs. In a cross-sectional analysis, numbers of circulating IgM memory B cells decline in the first 6 months and thereafter remained stable over many years.

## Methods

### Objectives

The study was designed to quantify the changes in B cell subsets in asplenic or hyposplenic patients in a population enrolled in a spleen registry.

### Participants

The Victorian Spleen Registry (VSR) was established to provide ongoing support for management of asplenic and hyposplenic subjects [7]. The registry was initially established within two teaching hospital networks in Victoria Australia (Bayside and Southern Health) that included specialist trauma centers (The Alfred and Dandenong Hospitals). The enrollments were initially biased toward incident splenectomy cases. Subsequently the registry was expanded to become a state-wide registry, that registered patients with a past history of splenectomy. At the time of the study there were 591 subjects enrolled.

Bloods from normal healthy controls were obtained from donors unselected for gender or age (n = 35) or as a population from the Australian Red Cross Blood Service (ARCBS) in Melbourne (n = 105) selected to equally represent donors across the adult age range from the 3rd to the 7th decade and included equal numbers of males and females. The initial control populations consisted of i] laboratory controls to establish the 3 colour flow assay (n = 15) and ii] normal laboratory donors used to establish the 5 colour assay (n = 20).

### Ethics

Patient consent to enroll with the spleen registry was given at the time that the referring practitioner completed the registration form. The referring practitioner had to indicate on the registration form (tick box) that the patient had been informed that relevant information and clinical details would be maintained by the Spleen Registry. If this box on the registration form was not ticked, the patient was not registered and the referring practitioner was contacted and asked to provide an amended form confirming consent prior to the patient being contacted by VSR staff. The operation of the VSR has been approved by the Human Ethics and Research Committee of Alfred Health for initial operation 3rd March 2003, and at the time of adoption of an opt-out recruitment strategy 30th Aug 2007 and for revised assessment form 4^th^ Dec 2007.

### Procedures

As part of routine assessment and monitoring of registry patients blood was collected from a subset of patients at varying times after splenectomy. Measurement of IgM memory B cells was performed on fresh blood samples at the same time as assessment for asplenic changes including presence of HJB on a routine blood film performed by the hematology laboratory service of the hospital. Most samples were collected at the time of enrollment in the spleen registry and in some incident splenectomy cases, blood was collected at routine follow-up within 12 months of splenectomy. The number of patients from the registry population, controls and non registry samples used in the different analyses in this study are shown in figure S1.

### Assays of B cell subsets including IgM memory B cells

**Figure 1:**
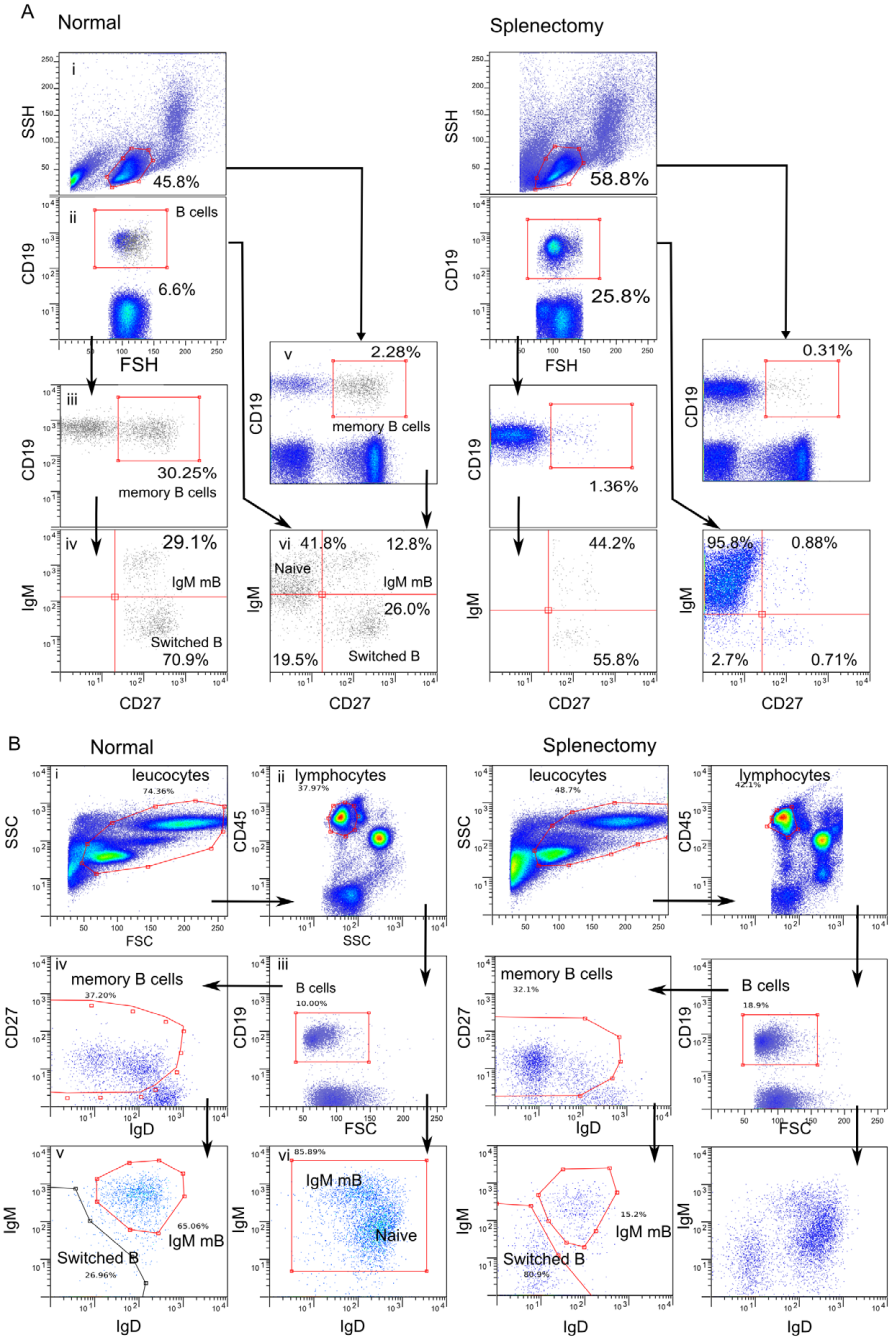
Representative dot plot profiles and gating strategies for 3 colour (A) and 5 colour (B) flow cytometric analysis of IgM memory B cells in normal and splenectomy patients. A. i) Lymphocytes were identified by forward (FSC) and side scatter(SSC), ii) B cells by CD19 expression (% B cells./lymph), iii) memory B cells by CD19, CD27 expression (% memory B cells/B cells), iv) IgM memory B as CD19+CD27+IgM+ (% IgM memory B cells/memory B cells) and switched memory B cells as CD19+CD27+IgM− (% switched memory B cells/memory B cells). Lymphocytes were also sequentially gated v) for CD19 CD27+ memory B cells (% memory B cells/lymph) and (vi) for CD27+ and IgM to identify IgM memory B cells [upper right quadrant] (% IgM memory B cells/B cells) and switched memory B cells [lower right quadrant] (switched memory B cells/B cells). Parameters used in subsequent quantitative analysis were B cells as a proportion of lymphocytes (ii), memory B cells as a proportion of B cells (iii) or total lymphocytes (v), and IgM memory cells as a proportion of B cells (vi). B. i) Blood leukocytes were identified by forward (FSC) and side scatter (SSC), ii) Lymphocytes selected by CD45 and log SSC, iii) B cells were identified by CD19 expression (% B cell/lymph) and iv) memory B cells were identified as CD19+CD27+IgD^lo^ cells (% memory B cells/lymph). v) memory B cells were identified as IgD^lo^IgM+ (% IgM memory B. memory B cells) and switched memory B cells as CD19+CD27+IgM−IgD− (% switched memory B cells/memory B cells). vi) lymphocytes gated as B cells and plotted for IgM and IgD are shown for comparison to identify the predominant naïve unswitched IgD^hi^ IgM+ B cell population.

A 3 colour flow cytometric assay using the surface markers CD19, CD27, and IgM was initially used to determine the number of IgM memory B cells in the registry patients. Peripheral blood mononuclear cells (PBMC) were prepared by standard methods using Ficoll-Paque density medium (GE Healthcare, Chalfont St. Giles, United Kingdom) or total white cells were prepared by adding 200 µl of whole blood to 3 ml of cold RBC lysis buffer (150 mM NH_4_Cl, 10 mM KHCO3 1 mM EDTA) for 5 min at room temperature. The white cells or PBMC were washed once with FACS wash (Phosphate Buffered Saline, 4 mM EDTA, 1% newborn calf serum (Cellgro, Invitrogen, San Diego, CA) and optimal concentration of CD19 ECD (Beckman Coulter), CD27 PE (Beckman Coulter, Fullerton, CA) and anti-IgM FITC (Caltag, Invitrogen, Carlsbad CA) antibodies were added for 40 minutes on ice. Cells were washed once in FACS wash (1% FCS and 4 mM EDTA in PBS) and fixed in 200 µl of 1% ultra-pure formalin (Pierce, Rockford, IL) in PBS. Analysis was performed within 24 hours using a FACScalibur 4 colour flow cytometer (Becton Dickinson, Palo Alto, CA). At least 200,000 events were collected and the strategy for gating to determine lymphocytes, B cells, memory B cells, IgM memory B cells and switched memory B cells is shown in figure 1A.

For a subset of patients, clinical samples and controls, the 3 colour assay was compared to a 5 colour assay using antibodies to CD45-ECD (Beckman Coulter), CD19-PE-Cy7(Beckman Coulter), CD27-PE-Cy5(Beckman Coulter), IgM-PE(Caltag) and IgD-FITC(Caltag). This 5 colour assay was adopted in the clinical laboratory and was used to establish a normal reference range from a larger healthy control population. Whole white cells were obtained by lysis of 100 µl of whole blood in 4 mls of RBC lysis buffer. Cells were washed once in FACS wash buffer and labeled with antibody for 25 minutes on ice, washed and fixed with 1% formalin. Cells were analyzed using an FC500 5 colour flow cytometer (Beckman Coulter). IgM memory B cells were sequentially gated as lymphocytes by forward scatter, side scatter, and expression of CD45, CD19, CD27, IgM and low IgD as shown in figure 1B.c

### Statistical methods

All spleen registry patients with B cell subset determination (n = 209) were compared to the normal reference population and changes with time analyzed using non-parametric statistical comparison of samples grouped by time using Mann Whitney U test. Only a small minority (n = 12) had more than one sequential assay and only one data point from each patient was included in any of the subgroups analyzed. The first sample was used for cross-sectional analysis by time and the last available sample was used for the comparison of B cell changes by cause of splenectomy.

Statistical tests were performed using the program R (v2.5.2) [34], or SAS version 9.1(SAS Institute, Cary, NC, USA). Measures of B cell sub-populations were assessed for normality and log transformed where appropriate. We assessed the effect of splenectomy, time after splenectomy, age and gender on B cell measures using the PROC Mixed procedure in SAS. The Mann Whitney U test was used to compare outcomes for populations according to the indication for splenectomy and for comparisons between populations at different times after splenectomy. Results from the mixed-effects model were presented as parameter estimates with standard errors or geometric means with 95% confidence intervals as appropriate. Correlations were performed using Pearson product moment correlation. Significance was considered with a probability <0.05. A Bonferroni correction was used where appropriate. Analysis of time to appearance of HJB was performed using the R package survival and the Kaplan-Meier estimator. The R package ROCR was used for the creation of Receiver Operating Curve (ROC) plots and analysis of accuracy, specificity, sensitivity and area under the curve.

## Results

### Demographics of patient population

Inclusion criteria were entry on the spleen registry, an available blood sample for a full blood count including blood film, and IgM memory B cells. Two groups of patients were analyses in this study. The first group of patients (n = 45) were incident cases that were enrolled during their hospital admission or during immediate follow-up and followed prospectively by the registry after splenectomy. These cases were all related to trauma and had routine hematological measures including blood film assessing for the presence of Howell Jolly Bodies from the time of admission. The second group consisted of 209 patients enrolled in the VSR at any time after splenectomy and were used in the cross-sectional analysis. Some demographics of this population are shown in Table 1A. This population was similar to the registry populations that were untested for B cell subsets as shown in Table 1B. The two populations were comparable in age, sex and indication for splenectomy. Within both groups, as expected, males were more common in the trauma group, older age more common where splenectomy was incidental to an associated surgical procedure,and malignancy was most frequent in surgical and hematological groups.

**Table 1:** Demographics of spleen registry populations.

A. Spleen registry population studied for IgM memory B cells						
	Number	Male (%)	Malignancy (%)	Deaths (%)	Age at registration (IQR)	<1 mth post-operation (%)
Trauma	96(45.9)	61(64)	0	4(4.1)	45.3(31.7–57.3)	33(46)
Haematological	40(19.1)	14(35)	10(25)	3(7.5)	51.6(42.1–63.1)	2 (5)
Surgical	41(19.6)	19(46.3)	16(39)	2(4.8)	67.9(57.5–75.5)	12(29)
Other	8(3.8)	2(25)	0	0	54.3(37.8–70.7)	2(25)
Hyposplenic	5(2.4)	2(40)	0	0	46.5(42.7–68.0)	NA
Embolization	19(9.1)	16(84.2)	0	4(21)	35.7(22.1–48.0)	17(89)
Total	209	114(54.5)	26(12)	13(6)	50.8(36.2–63.7)	66(32)

B. Spleen registry population not available for IgM memory B cells						
Trauma	154(40.3)	108(70.1)	0	16(10.4)	43.5(27.5–54.4)	71(46)
Haematological	116(30.4)	50(43.1)	30(25.9)	7(6.0)	50.6(36.7–61.2)	23 (20)
Surgical	61(16.0)	33(54.1)	33(54.1)	6(9.8)	46.7(52.6–73.3)	26(43)
Other	34(8.9)	15(44.1)	3(8.8)	0	48.2(36.6–58.2)	8(24)
Hyposplenic	7(1.8)	2(28.6)	0	0	38.9(16.1–52.9)	NA
Embolization	10(2.6)	5(50%)	0	4(40)	28.1(23.4–40.1)	7(70)
Total	382	213(56)	36(17)	33(9)	47.7(33.5–65.1)	135(35.3)
		*NS*	*NS*	*NS*	*NS*	*NS*

Demographics of the patients with available blood test for IgM memory B cells (A) and those without an available result (B). The gender distribution (male), frequency of malignancy and death, and frequency of early registration (<1month post operation) were compared by Chi squared test. The age at registration was compared by Wilcoxon test and is shown as median and interquartile range (IQR). NS = not significant, NA = not applicable.

### Comparison of assays for IgM memory B cells

**Figure 2:**
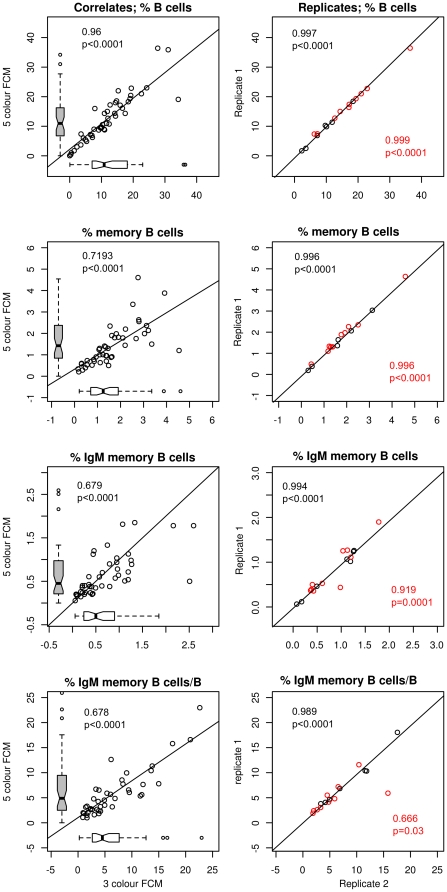
Comparison of 3 colour and 5 colour assays. Distribution of normal laboratory controls used for 3 colour (n = 15) and 5 colour assays (n = 20) is shown. These 2 control populations were composed of different sets of laboratory controls recruited at different times. Left column indicates the correlations between results for a cohort of patients assayed as fresh samples by both 3 colour and 5 colour assays. The right column represents replicates in each assay with 3 colour assay shown in black and the 5 colour assay in red. Correlation was determined by Pearson product moment correlation for 3 colour (black) and 5 colour (red) analyses. Box-plot show the median as the horizontal line in the middle of the box, 25% as the lower end of the box 75% as the upper end and whiskers show the range.

Initial assays for IgM memory B-cells was performed using a 3 colour flow cytometric analysis as shown in figure 1. The marker CD19 was used to identify B cells and CD27 to identify the memory cell subset. IgM memory B cells were identified as cells expressing IgM, CD27, and CD19 within the lymphocyte population identified by forward and side scatter characteristics. The second assay method used for measurement of IgM memory B cells used 5 colour analysis including CD45 to more accurately exclude any residual RBCs from the analysis (Figure 1B). Comparison between the 3 colour assay and the 5 colour total white cell assays are shown in figure 2 where a sample set were analyzed by both assays. Reference ranges for the different control groups were comparable and there was good correlation between samples tested on both assays.

### Changes in IgM memory B cell numbers after splenectomy

**Figure 3:**
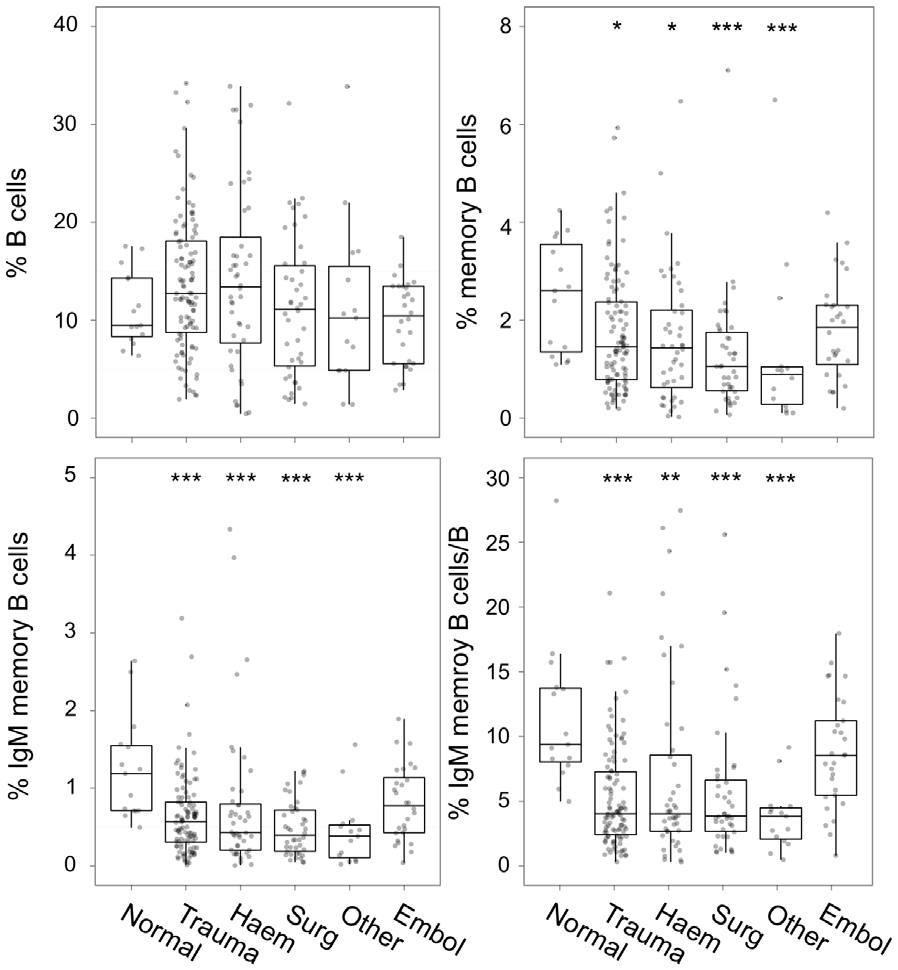
Distribution of B cell subsets in normal and hyposplenia individuals by indication for splenectomy. Subjects were classified by indication for splenectomy and compared to normal laboratory controls (n = 15). Normal = normal controls, Trauma = splenectomy related to trauma, Haem = haematological disease, Surg = splenectomy incidental to abdominal surgery, Embol = splenic embolization. Box-plots show the median as the horizontal line in the middle of the box, 25% as the lower end of the box 75% as the upper end and whiskers show the range. Bonferroni corrected p values determined by Wilcoxon non-parametric statistic * <0.05, ** <0.001, *** <0.0001 for comparisons to normal controls.

A normal laboratory control population (n = 15) was compared to patients from the spleen registry (n = 209) using the 3 colour assay. Splenectomy was found to be associated with a decreased proportion of both the total memory cell population and the IgM memory cell population but not total B cell numbers (Figure 3). We then analyzed patients from the spleen registry according to the underlying disease leading to splenectomy (Figure 3) to determine if there was any effect of the underlying pathological process on the distribution of B cells subsets after splenectomy. A significant reduction in all memory B cell subsets was found for splenectomized patients compared to the normal reference population (p<0.01 for memory B cells/lymphocyte, p<0.001 for IgM memory B cells/lymphocytes, p<0.00001 for IgM memory B cells/B cells). There was no effect of underlying pathology on the proportion of total B cells, memory B cells or IgM memory B cells. Patients with a history of splenic artery embolization for traumatic spleen injury did not differ significantly from normal controls in the proportions of the B cell sub-populations (uncorrected p values = 0.5 for B cells, 0.1 for memory B cells, 0.07 for IgM memory B cells, and 0.15 for IgM memory B cells/B cells).

### Kinetics of splenectomy related change in B cells and hematological parameters

To further determine if the differences between patients following splenectomy or splenic artery embolization was due to difference in the time at which the sample was collected relative to the time of splenectomy or embolization, we next examined the kinetics of changes in memory B cells after splenectomy using a cross-sectional analysis of the splenectomy population. We used the first sample from any patient with repeat assays (n = 12).

**Figure 4:**
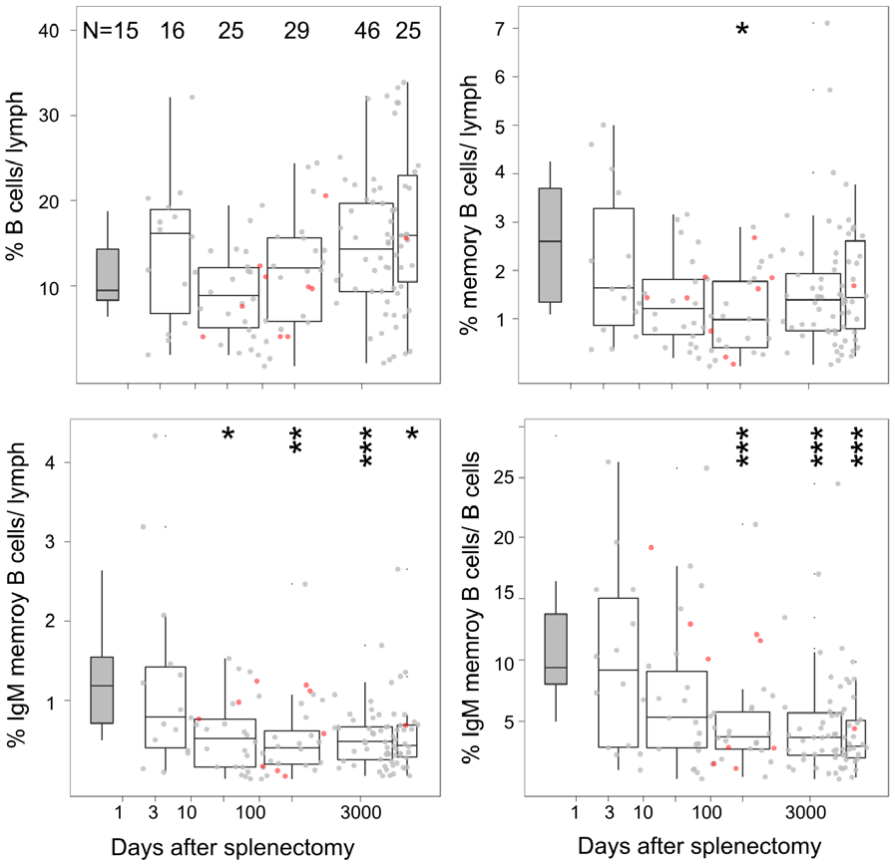
Kinetics of changes in B cell subsets after splenectomy. The time in days post-splenectomy for registry patients was determined from date of splenectomy to date of sample collection and plotted against proportion of lymphocytes represented by B cell numbers (i), memory B cells (ii), and IgM memory B cells (iii) and IgM memory B cells as proportion of total B cells (iv). Data covering periods 1–10 days, 10–100 days, 100–1000 days, 10000–100001 days and >10000 days were pooled for analysis. Only the analysis at the first assessment for each patient is shown and repeat measures shown in red were excluded from statistical analysis. Box-plot show the median as the horizontal line in the middle of the box, 25% as the lower end of the box 75% as the upper end and whiskers show the range. Outliers are omitted from the box-plots. The grey box-plot represents the normal reference range for the assay. Bonferroni corrected p values determined by Wilcoxon non-parametric statistic * <0.05, ** <0.001, *** <0.0001 for comparisons to normal controls.

We assumed that the underlying indication for the splenectomy had no effect on the decay of IgM memory B cells and demonstrated an apparent exponential decline in IgM memory B cells within the first 100–150 days but stable levels beyond this time (Figure 4). The selected trauma group showed the same pattern of decrease shown in the total splenectomy population (corrected p values = 0.008, 0.0128, 0.008 for the 100–1000, 1000–10000 and >10000 days post splenectomy).

**Figure 5:**
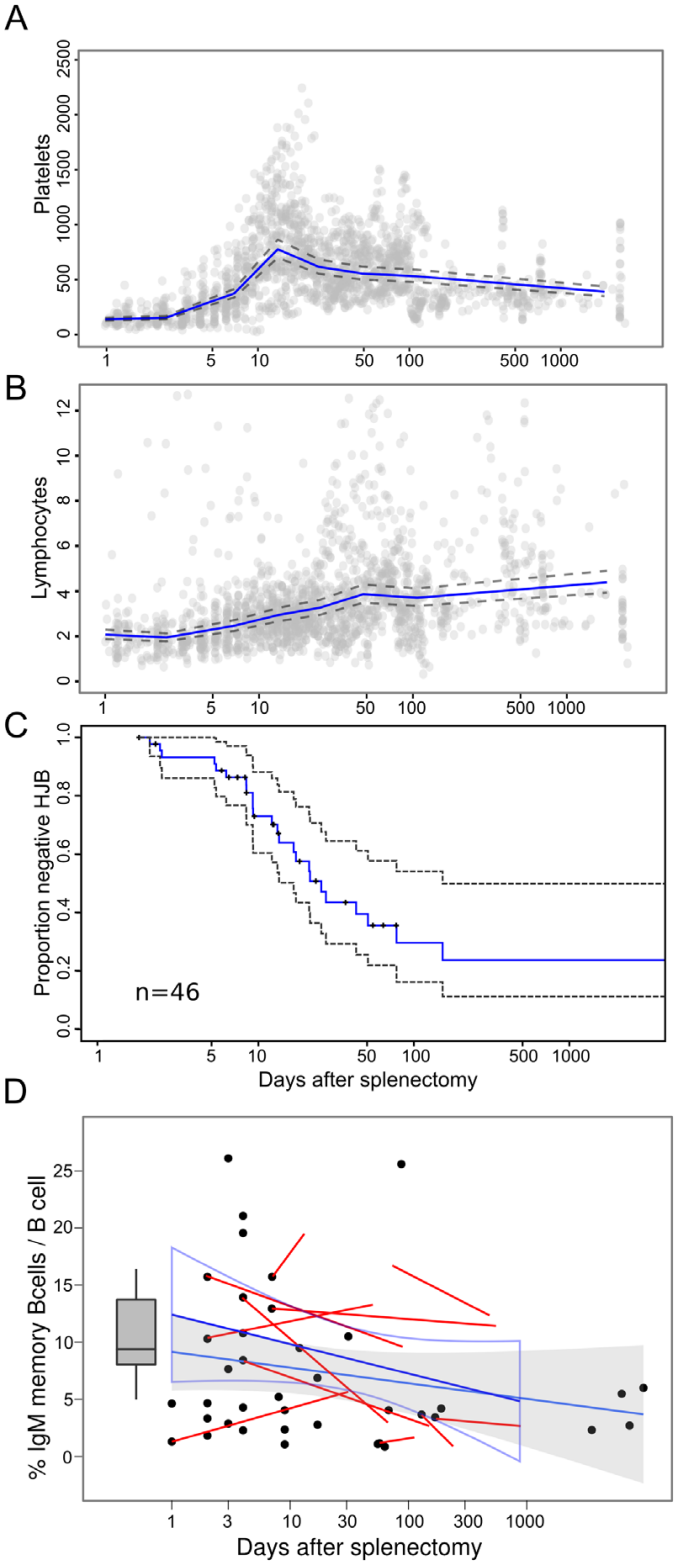
Kinetics of change in haematological parameters and IgM memory B cells after splenectomy. Incident cases of splenectomy (n = 51) were examined for changes in haematological parameters on sequential blood films and automated lymphocyte and platelet counts. A] Changes in platelet count post splenectomy. The data points and the geometric mean and 95% confidence intervals for repeat measure ANOVA for observations divided into deciles by time are shown on a log time scale. B] Changes in lymphocyte numbers post splenectomy. Similar analysis as shown in A. C] Kaplan Meier plot of rate of appearance of asplenic changes including HJB on blood film. The analysis was performed on a subset of patients (n = 45) with absence of HJB on the first analysis. The 95% confidence intervals for the function are shown by grey dashed lines. Median time to appearance of HJB was 25 days. D] Changes in IgM memory B cells as proportion of B cells with time. Relationship between IgM memory B cells and time post splenectomy was determined in the subset shown in C with available IgM assay (n = 42). Single values are plotted as a dot-plot and sequential data (n = 12) are shown as line plot overlay. A linear regression and 95% confidence interval (grey) is fitted for the single data point and the sequential samples (open box). The grey histogram indicates the range for the normal controls.

We next examined changes in lymphocyte and platelet numbers over time in a subset of patients followed from the time of splenectomy (Figure 5A,B). There was an increase in platelets and lymphocytes with a peak in platelets at 20 days and lymphocytes at approximately 50 days. We then examined the kinetics of appearance of HJB on blood film. The appearance was rapid with 50% of subjects having HJB present by 25 days (Figure 5C). We then analysed the changes in IgM memory B cells in this group of subjects (figure 5D). The pattern of loss in this population was similar to that found in the cross-sectional analysis of the larger population. In the patients with measurement of IgM memory B cells at 2 time points the decline was similar to those with single measurement.

### IgM memory B cells as predictors of loss of splenic tissue

**Figure 6:**
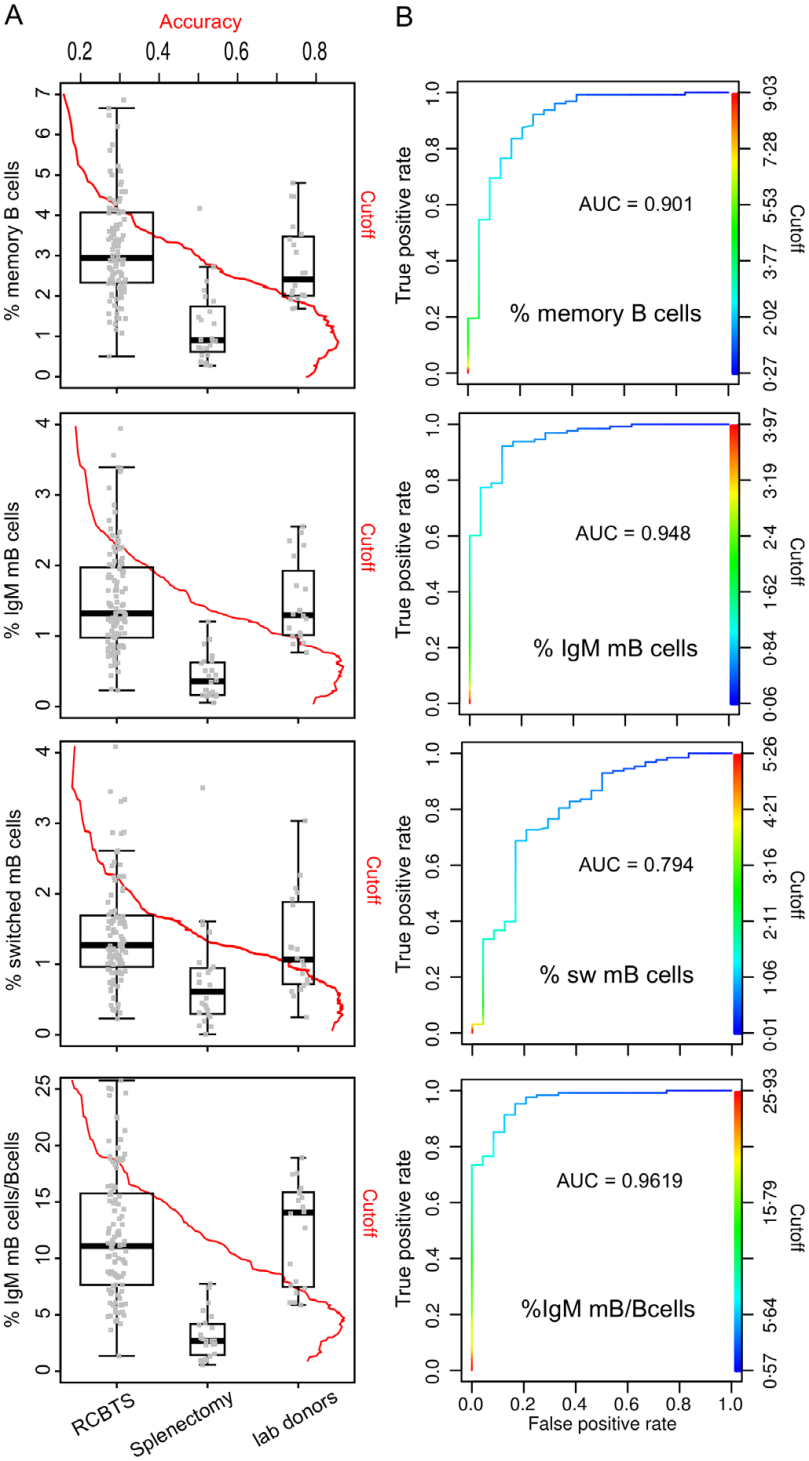
Reference ranges for B cell subsets in normal donors and asplenic patients. A] Five colour flow cytometry was used to measure the number of CD19+ B cells, CD27+ memory B cells, IgM memory B cells and CD27+IgM− switched B cells. Normal laboratory donors (lab donors n = 20), Red Cross Blood Transfusion Service donors (RCBTS, n = 105) and samples from patients at least 12 months after splenectomy (splenectomy, n = 36) were tested and results expressed as proportion of lymphocytes or of B cells. box-plots show the median as the horizontal line in the middle of the box, 25% as the lower end of the box 75% as the upper end and whiskers show the range. B] Receiver operator curves were used to analyse each of the 4 parameters including CD19+CD27+ memory B cells, IgM memory B cells, CD27+IgM−IgD− switched memory B cells, and IgM memory B cells as proportion of B cells as discriminators between normal and asplenic individuals. Area under the curve for each plot is shown in B. The relationship of accuracy against cut-off is plotted in A with the box-plots.

To establish the reference range and to determine the predictive value of IgM memory B cell measurement, we examined a population of normal blood donors using 5 colour flow cytometry. The reference population (n = 105) was normal blood bank donors. Blood from the normal donors was processed on the day of venepuncture and the different B cell populations identified by flow cytometry. To determine the performance of the IgM memory B cell assays as markers of splenectomy a comparison population of patients (n = 24) more than 2 years after splenectomy was used (Figure 6). Using the optimal cut-off for IgM memory B cells as a proportion of total B cells of 4.53, we found a true positive rate of 95% and false positive rate of 20%. Other parameters including total memory B cells, and switched memory B cells had lower AUC and were less predictive value for splenic loss.

### Age related changes in B cells in normal donor populations

Since there was a trend to increased levels of IgM memory B cells in our laboratory control populations compared to the blood bank donor population we sought to analyze the donors by age and gender. We compared these populations and showed no significant decrease in IgM memory B cells by age in the healthy blood donor population but a decrease in B cells and memory B cells with age in the splenectomy population (Table 2). There was no effect of gender. We further analyzed the contribution of age at splenectomy and time of follow-up to determine if there was evidence of a difference in IgM memory cells persistence where splenectomy was performed at an early age. A significant negative correlation was found with age at splenectomy and B and memory B cells but not in the proportion of IgM memory B cells (figure S2). No correlation was found for changes in memory B cells or B cells by time of follow-up.

**Table 2:** Effects of age and gender on B cell subsets after splenectomy in mixed model analysis.

	Age			Male gender		
	logEst	logStderr	logPvalue	logEst	logStderr	logPvalue
% B cells	−0.006	0.002	0.001	−0.129	0.11	0.24
% mB cells	−0.010	0.002	<0.001	−0.059	0.13	0.65
% IgM mB cells	−0.011	0.002	<0.001	−0.017	0.139	0.902
% IgM mB/B cells	−0.005	0.002	0.014	0.121	0.126	0.339
% IgM mB/mB	0.0	0.001	0.714	0.074	0.065	0.259

Data was log transformed to normalize the distribution and the effects of age and gender on B cell subpopulations were determined. Populations included B cell (% B cells), memory B cells (% mB cells) and IgM memory B cells (IgM mB cells) as a proportion of lymphocytes, IgM memory B cells as a proportion of B lymphocytes (% IgM mB/B cells) and IgM memory B cells as a proportion of memory B cells (% IgM mB/mB).

### Clinical associations with changes in IgM memory B cell numbers

We looked for association between memory B cell numbers and infectious complications of splenectomy using a patient survey. Of registry patients with available IgM memory B cell determination, 152 responded to a survey of infection risk in the preceding 12 months (response rate of 73%, table S1). Patients with IgM memory B cells above the cut-off were more likely close to the time of splenectomy and less likely to have detectable HJB but these differences where not significant when the analysis was restricted to those where IgM memory B cells were measured more than 200 days after splenectomy. No association of low IgM memory B cells with infection, antibiotic use or hospitalization with infection was found. No registered patients experienced OPSI during the study. The registry population in the survey had high levels of compliance with annual influenza vaccination (71%), and prophylactic or emergency supply of antibiotics (63%).

## Discussion

In this study we examined the changes in B cell subpopulations in a cohort of patients enrolled in a spleen registry. The main finding in these populations was a significant decrease in total CD27+ memory B cells and in the IgM+IgD+CD27+IgM memory B cells compared to normal controls. There was no association between the number in the B cell subsets and the underlying indication for splenectomy; trauma, haematological disease, incidental surgical procedure or other medical disease. The decline in IgM memory B cells after splenectomy was slower than for changes in blood film parameters and reached a stable nadir within 6 months after splenectomy. In contrast, appearance of other markers for splenic dysfunction was more rapid with median time to appearance of HJB at 25 days, a peak in thrombocytosis at 20 days and a peak in post-splenectomy lymphocytosis at 50 days. These findings suggested that the half-life of IgM memory B cells in blood may be longer than for changes in other indicators of splenic RBC clearance. Further it suggests that measurement of IgM memory cells is not a good indicator of the presence of splenic tissue until 6 months after splenectomy.

This is the largest study of memory B cell subsets in splenectomized patients with varying indications for splenectomy. A number of smaller studies have examined IgM memory B cells in splenectomized subjects [13], [17], [35]. Initial work that examined the relationship between IgM memory B cell numbers and splenectomy suggested that there was complete loss of IgM memory B cells but the sample size was small (n = 11) [13] and included subjects late after splenectomy(0.5–9 years). Another study showed that B cell populations in the blood of asplenic children (n = 10) was reduced but not absent [14]. In a comparison of memory B cells, IgM memory B cells and antibody responses to vaccination it was found that there was no difference in antibody responses between controls and those with splenectomy for spherocytosis [17] but decreased IgM responses and reduced IgM memory B cells in those with splenectomy for Idiopathic Thrombocytopenic Purpura (ITP). This difference may be due to the differences in timing of splenectomy in these populations or to persistence of some alternative reservoir of IgM memory B cells after splenectomy at an early age.

Changes in IgM memory B cells with age have been reported [17], with one study reporting dramatic decline in memory B cells with advancing age [36]. In normals we found no significant relationship between age and total B cells, memory B cells, and IgM memory B cells. After splenectomy we found age related decrease in B cells and memory B cells but no significant correlation with IgM memory B cells as proportion of memory B cells suggesting that splenectomy related changes affect B cell homeostasis beyond IgM memory cells alone. Although there are early changes of lymphocytosis and thrombocytosis associated with splenectomy we found a delayed decline in IgM memory B cells with a long period with stable low levels after an initial apparently exponential decline. This suggests either slow turnover or clearance from the blood after their splenic source of origin is removed and that there are alternative sources of the IgM memory B cells accounting for low but persistent numbers. Previous research in man has shown a faster turnover of CD27+ memory compared to naïve B cells [37] but this study did not divide the CD27+ memory B cells populations by IgD or IgM expression to identify IgM memory cells and switched memory B cells and was not performed in asplenic subjects [37]. The IgM memory cell population may include distinct sub-populations with different rates of turnover. B cell receptor sequencing data indicates a proportion of the IgM expressing memory B cells may derive from the germinal center reaction [38], [39]. Murine marginal zone IgM+ memory cells have a slow turnover and are restricted to the spleen [32] however one recent study has addressed the question of loss of IgM memory B cells after splenectomy in patients with ITP and shown a more rapid loss of IgM memory B cells than switched memory B cells [18]. Our cross-sectional data on kinetics of loss does agree with these authors' findings of decline of IgM memory B cells within 8 months of splenectomy to a stable level up to more than 2 years [18]. This persistent reduced but stable level after the initial decline is consistent with a pool of IgM memory cells with long half life or more likely with IgM memory B cell populations derived from germinal centre reactions outside the spleen. Both possibilities would reduce the value of IgM memory B cell assays as biomarkers of functional splenic tissue.

Age related changes may be important in the interpretation of the reference populations. An increase in memory cells with age has been reported with approximately 30% memory B cells in young subjects rising to 45% in elderly [37]. Sources of IgM memory B cells other than from the spleen [38] may also affect the rate of decay in IgM memory B cells after splenectomy at a young age is suggested by the observations of persistence of IgM memory B cells after splenectomy for spherocytosis compared to ITP [17]. We were unable to differentiate between different sources of IgM memory B cells but have demonstrated that in the early post operative period both memory B cells and IgM memory B cells are normal.

The clinical significance of loss of IgM memory B cells in humans has only been established indirectly through the association of low IgM memory cells with complications of asplenia. The risk of OPSI has been attributed to both reduced clearance of bacteria as evident by presence of abnormal red cells indicated by presence of HJB and the loss of IgM memory B cells which provide innate immune responses [13]. The assumption that only marginal zone IgM memory B cells serve an innate defence role however has been questioned [40]. Recent work has suggested that the CD27+ B cells which will include the IgM memory cell population may be divided by CD43 expression into a B1 cell population that is responsible for production of natural antibody and IgM production [41], functions attributed to IgM memory B cells [13]. In mouse CD5+ B1 cells (B1a) although not exclusively localized to the spleen do depend on the spleen for production and maintenance [42]. There is a need for further study to directly determine the relationship between vaccine responses to polysaccharide vaccines according to the levels of IgM memory B cells including both germinal centre derived IgM memory B cells and B1 cells in both asplenic and normal individuals.

Our data indicating high false negative rate suggests that conventional measurement of IgM memory B cells alone may not be useful in identifying asplenia or in identification of subjects at risk for OPSI. It may however supplement the detection of HJB in the identification of patients at risk for hyposplenism for further assessment of splenic function by other methods [43].

This study has some limitations: i] we did not assess for function of the populations of IgM memory B cells. Currently it is considered that circulating IgM memory B cells in addition to representing a) circulating splenic marginal zone B cells may include b) germinal center B cells from various sites that have not been subject to class switch [39] and may be functionally similar to switched memory B cells, and c) B1 cells [41]. Cells with the phenotype of IgM memory B cells may have splenic origins by a distinct pathway that is critical for early response to T cell independent polysaccharide antigens [44]–[46]. This is supported by recent B cell receptor sequencing data [38], [39] and by murine data on pyk2 signaling pathway [44]. Further analysis of functional changes in IgM memory B cells after splenectomy will be critical in determining the contribution of T cell independent IgM memory B cell subpopulations to protection from OPSI. ii] Clinical events are uncommon in the spleen registry population as we have recently reported [47] and correlation between numbers within subpopulations of IgM memory B cells and clinical events will require large prospective studies on such populations. iii] In this study we had limited longitudinal data as IgM memory B cells were usually assayed on only one occasion around the time of enrollment in the registry. The analysis of kinetic of IgM memory cell loss that we performed may have been confounded by a survival bias. iv] Two different assays were used in the study. Initially we used a simple 3 colour cytometric assay using CD19, CD27 and IgM that was later replaced by a 5 colour assay including CD45 and IgD that has been adopted by the clinical laboratory. The reference ranges for these assays were similar and the samples that were assayed by both methods were found to correlate. We have also compared our reference ranges to other published studies of IgM memory B cells in different diseases using different methods [13], [17], [19], [25], [48]–[52] and found a comparable range for the controls [figure S3].

In summary we showed in a large spleen registry population that there was a significant decrease in IgM memory B cells in splenectomized subjects and that this was not related to the indication for splenectomy. Our data suggest that measurement of IgM memory B cells may be insensitive in the identification of patient with functional splenic tissue and will have a limited role in stratification of risk of OPSI or identifying risk of less severe infections in splenectomized populations.

## Supporting Information

Figure S1 **Patient samples used in the study.** IgM memory B cell assays were performed at the time of entry for 209 of the the registry patients (n = 591). This populations was used to determine relationship between cause for splenectomy and IgM memory B cells and in the analysis of changes in IgM memory B cells with time following splenectomy. A subset of “incident” patients was used to determine haematological changes in comparison to IgM memory B cells changes. 51 patients had haematological data from the time of splenectomy of which 45 had an initial blood film that did not show HJB and follow-up blood films. Of these 42 had measurement of IgM memory B cells including 12 with sequential measures. Two assays for IgM memory B cells were used in this study; an initial 3 colour analysis that was compared to a normal control population (n = 15) and later a 5 colour assay that was compared to a normal control population (n = 20). These 2 assays were directly compared for 48 samples including some non-registry patient samples and registry patients. The reference values for this assay were established using a normal blood bank donor population that was selected to cover the adult age range and equally represent male and females. This was compared by ROC analysis against a splenectomy population that was more than 1 year post-splenectomy (n = 24). Data was obtained from patient survey of compliance with spleen registry recommendations. The subset of patients tested for IgM memory B cells (n = 152) was then used to determine relationship between the parameter “IgM memory B cells/B cells” and measures of compliance and indicators of infection.
(TIF)

Figure S2 **Correlation of changes in B cell subsets with age at splenectomy and time post splenectomy.** Patients after splenectomy were analyzed for changes in proportion of B cells, memory B cells, IgM memory B cells with age (left column). The variable of changes in B cell with age was resolved into comparisons of changes in B cells with time since splenectomy (middle column) and changes with age at the time of splenectomy (right column). The Pearson correlation and p values for the correlations are shown above each plot.
(TIF)

Figure S3 **Comparison of reference range from the current study with other studies of IgM memory B cells and B cell subsets.** The summary data for healthy control populations were taken from published studies and plotted as mean and SD, median and IQR (blue) or median and range (red). The size of the mean/median symbol is proportional to the log of the number of subjects in the reference population. Each cell type is shown as proportion of B cells (B) or of total lymphocytes (L).
(TIF)

Table S1 **IgM memory B cells as a predictor of infectious complications after splenectomy.** Patients were divided into 2 groups based on IgM memory B cell levels above (>cutoff) or below (<cutoff) the cutoff determined by the ROC analsysis shown in fig. 6. The 2 groups where then compared for parameters determined from the patient survey. The initial analysis (upper panel) found significant association with absent HJB and had a nuber of patients at an early time post splenectomy. The analysis was repeated after exclusion of subjects where the IgM memory B cells had been determine less than 200 days after splenectomy.
(DOC)

## References

[1] Waghorn DJ (2001). Overwhelming infection in asplenic patients: current best practice preventive measures are not being followed.. J Clin Pathol.

[2] Cullingford GL, Watkins DN, Watts AD, Mallon DF (1991). Severe late postsplenectomy infection.. Br J Surg.

[3] Okabayashi T, Hanazaki K (2008). Overwhelming postsplenectomy infection syndrome in adults - a clinically preventable disease.. World J Gastroenterol.

[4] Waghorn DJ, Mayon-White RT (1997). A study of 42 episodes of overwhelming post-splenectomy infection: is current guidance for asplenic individuals being followed.. J Infect.

[5] Gaston MH, Verter JI, Woods G, Pegelow C, Kelleher J (1986). Prophylaxis with oral penicillin in children with sickle cell anemia.. A randomized trial N Engl J Med.

[6] Castagnola E, Fioredda F (2003). Prevention of life-threatening infections due to encapsulated bacteria in children with hyposplenia or asplenia: a brief review of current recommendations for practical purposes.. Eur J Haematol.

[7] Spelman D, Buttery J, Daley A, Isaacs D, Jennens I (2008). Guidelines for the prevention of sepsis in asplenic and hyposplenic patients.. Intern Med J.

[8] Price VE, Dutta S, Blanchette VS, Butchart S, Kirby M (2006). The prevention and treatment of bacterial infections in children with asplenia or hyposplenia: practice considerations at the Hospital for Sick Children, Toronto.. Pediatr Blood Cancer.

[9] El-Alfy MS, El-Sayed MH (2004). Overwhelming postsplenectomy infection: is quality of patient knowledge enough for prevention.. Hematol J.

[10] Ramachandra J, Bond A, Ranaboldo C, Cullis J (2003). An audit of post-splenectomy prophylaxis–are we following the guidelines.. Ann R Coll Surg Engl.

[11] Babiker MA (1986). Compliance with penicillin prophylaxis by children with impaired splenic function.. Trop Geogr Med.

[12] Bitarães EL, Oliveira BM de, Viana MB (2008). Compliance with antibiotic prophylaxis in children with sickle cell anemia: a prospective study.. J Pediatr (Rio J).

[13] Kruetzmann S, Rosado MM, Weber H, Germing U, Tournilhac O (2003). Human immunoglobulin M memory B cells controlling Streptococcus pneumoniae infections are generated in the spleen.. J Exp Med.

[14] Weller S, Braun MC, Tan BK, Rosenwald A, Cordier C (2004). Human blood IgM “memory” B cells are circulating splenic marginal zone B cells harboring a prediversified immunoglobulin repertoire.. Blood.

[15] Agematsu K, Futatani T, Hokibara S, Kobayashi N, Takamoto M (2002). Absence of memory B cells in patients with common variable immunodeficiency.. Clin Immunol.

[16] Carsetti R, Rosado MM, Donnanno S, Guazzi V, Soresina A (2005). The loss of IgM memory B cells correlates with clinical disease in common variable immunodeficiency.. J Allergy Clin Immunol.

[17] Wasserstrom H, Bussel J, Lim LC-L, Cunningham-Rundles C (2008). Memory B cells and pneumococcal antibody after splenectomy.. J Immunol.

[18] Martinez-Gamboa L, Mei H, Loddenkemper C, Ballmer B, Hansen A (2009). Role of the spleen in peripheral memory B-cell homeostasis in patients with autoimmune thrombocytopenia purpura.. Clin Immunol.

[19] Hart M, Steel A, Clark SA, Moyle G, Nelson M (2007). Loss of discrete memory B cell subsets is associated with impaired immunization responses in HIV-1 infection and may be a risk factor for invasive pneumococcal disease.. J Immunol.

[20] Di Sabatino A, Rosado MM, Ciccocioppo R, Cazzola P, Morera R (2005). Depletion of immunoglobulin M memory B cells is associated with splenic hypofunction in inflammatory bowel disease.. Am J Gastroenterol.

[21] Bouts AHM, Davin JC, Krediet RT, Monnens LAH, Nauta J (2004). Children with chronic renal failure have reduced numbers of memory B cells.. Clin Exp Immunol.

[22] Di Sabatino A, Rosado MM, Miele L, Capolunghi F, Cazzola P (2007). Impairment of splenic IgM-memory but not switched-memory B cells in a patient with celiac disease and splenic atrophy.. J Allergy Clin Immunol.

[23] Di Sabatino A, Carsetti R, Rosado MM, Ciccocioppo R, Cazzola P (2004). Immunoglobulin M memory B cell decrease in inflammatory bowel disease.. Eur Rev Med Pharmacol Sci.

[24] Sánchez-Ramón S, Radigan L, Yu JE, Bard S, Cunningham-Rundles C (2008). Memory B cells in common variable immunodeficiency: clinical associations and sex differences.. Clin Immunol.

[25] D'Orsogna LJ, Krueger RG, McKinnon EJ, French MA (2007). Circulating memory B-cell subpopulations are affected differently by HIV infection and antiretroviral therapy.. AIDS.

[26] Subramaniam K, Metzger B, Hanau LH, Guh A, Rucker L (2009). IgM(+) memory B cell expression predicts HIV-associated cryptococcosis status.. J Infect Dis.

[27] Morrow M, Valentin A, Little R, Yarchoan R, Pavlakis GN (2008). A splenic marginal zone-like peripheral blood CD27+B220- B cell population is preferentially depleted in HIV type 1-infected individuals.. AIDS Res Hum Retroviruses.

[28] Titanji K, De Milito A, Cagigi A, Thorstensson R, Grützmeier S (2006). Loss of memory B cells impairs maintenance of long-term serologic memory during HIV-1 infection.. Blood.

[29] Martin F, Oliver AM, Kearney JF (2001). Marginal zone and B1 B cells unite in the early response against T-independent blood-borne particulate antigens.. Immunity.

[30] Boes M, Prodeus AP, Schmidt T, Carroll MC, Chen J (1998). A critical role of natural immunoglobulin M in immediate defense against systemic bacterial infection.. J Exp Med.

[31] Baxendale HE, Johnson M, Stephens RCM, Yuste J, Klein N (2008). Natural human antibodies to pneumococcus have distinctive molecular characteristics and protect against pneumococcal disease.. Clin Exp Immunol.

[32] Pillai S, Cariappa A (2009). The follicular versus marginal zone B lymphocyte cell fate decision.. Nat Rev Immunol.

[33] Song H, Cerny J (2003). Functional heterogeneity of marginal zone B cells revealed by their ability to generate both early antibody-forming cells and germinal centers with hypermutation and memory in response to a T-dependent antigen.. J Exp Med.

[34] Ihaka R, Gentleman R (1996). R: A language for data analysis and graphics.. Journal of Computational and Graphical Statistics.

[35] Carsetti R, Pantosti A, Quinti I (2006). Impairment of the antipolysaccharide response in splenectomized patients is due to the lack of immunoglobulin M memory B cells.. J Infect Dis.

[36] Shi Y, Yamazaki T, Okubo Y, Uehara Y, Sugane K (2005). Regulation of aged humoral immune defense against pneumococcal bacteria by IgM memory B cell.. J Immunol.

[37] Macallan DC, Wallace DL, Zhang Y, Ghattas H, Asquith B (2005). B-cell kinetics in humans: rapid turnover of peripheral blood memory cells.. Blood.

[38] Wu Y-C, Kipling D, Leong HS, Martin V, Ademokun AA (2010). High-throughput immunoglobulin repertoire analysis distinguishes between human IgM memory and switched memory B-cell populations.. Blood.

[39] Seifert M, Küppers R (2009). Molecular footprints of a germinal center derivation of human IgM+(IgD+)CD27+ B cells and the dynamics of memory B cell generation.. J Exp Med.

[40] Tangye SG, Good KL (2007). Human IgM+CD27+ B Cells: Memory B Cells or “Memory” B Cells?. J Immunol.

[41] Griffin DO, Holodick NE, Rothstein TL (2011). Human B1 cells in umbilical cord and adult peripheral blood express the novel phenotype CD20+CD27+CD43+CD70−.. J Exp Med.

[42] Wardemann H, Boehm T, Dear N, Carsetti R (2002). B-1a B cells that link the innate and adaptive immune responses are lacking in the absence of the spleen.. J Exp Med.

[43] de Porto APNA, Lammers AJJ, Bennink RJ, Ten Berge IJM, Speelman P (2010). Assessment of splenic function.. Eur J Clin Microbiol Infect Dis.

[44] Guinamard R, Okigaki M, Schlessinger J, Ravetch JV (2000). Absence of marginal zone B cells in Pyk-2-deficient mice defines their role in the humoral response.. Nat Immunol.

[45] Martin F, Kearney JF (2002). Marginal-zone B cells.. Nat Rev Immunol.

[46] Zandvoort A, Timens W (2002). The dual function of the splenic marginal zone: essential for initiation of anti-TI-2 responses but also vital in the general first-line defense against blood-borne antigens.. Clin Exp Immunol.

[47] Denholm JT, Jones PA, Spelman DW, Cameron PU, Woolley IJ (2010). Spleen registry may help reduce the incidence of overwhelming postsplenectomy infection in Victoria.. Med J Aust.

[48] Warnatz K, Denz A, Dräger R, Braun M, Groth C (2002). Severe deficiency of switched memory B cells (CD27(+)IgM(−)IgD(−)) in subgroups of patients with common variable immunodeficiency: a new approach to classify a heterogeneous disease.. Blood.

[49] Piqueras B, Lavenu-Bombled C, Galicier L, Bergeron-van der Cruyssen F, Mouthon L (2003). Common variable immunodeficiency patient classification based on impaired B cell memory differentiation correlates with clinical aspects.. J Clin Immunol.

[50] Ferry BL, Jones J, Bateman EA, Woodham N, Warnatz K (2005). Measurement of peripheral B cell subpopulations in common variable immunodeficiency (CVID) using a whole blood method.. Clin Exp Immunol.

[51] Vodjgani M, Aghamohammadi A, Samadi M, Moin M, Hadjati J (2007). Analysis of class-switched memory B cells in patients with common variable immunodeficiency and its clinical implications.. J Investig Allergol Clin Immunol.

[52] Berglund LJ, Wong SWJ, Fulcher DA (2008). B-cell maturation defects in common variable immunodeficiency and association with clinical features.. Pathology.

