# Supplementary figures and images for: Splenectomy Associated Changes in IgM Memory B Cells in an Adult Spleen Registry Cohort
Source: PLoS One. 2011 Aug 4;6(8):e23164. doi: 10.1371/journal.pone.0023164 (PMC3150402; doi:10.1371/journal.pone.0023164)

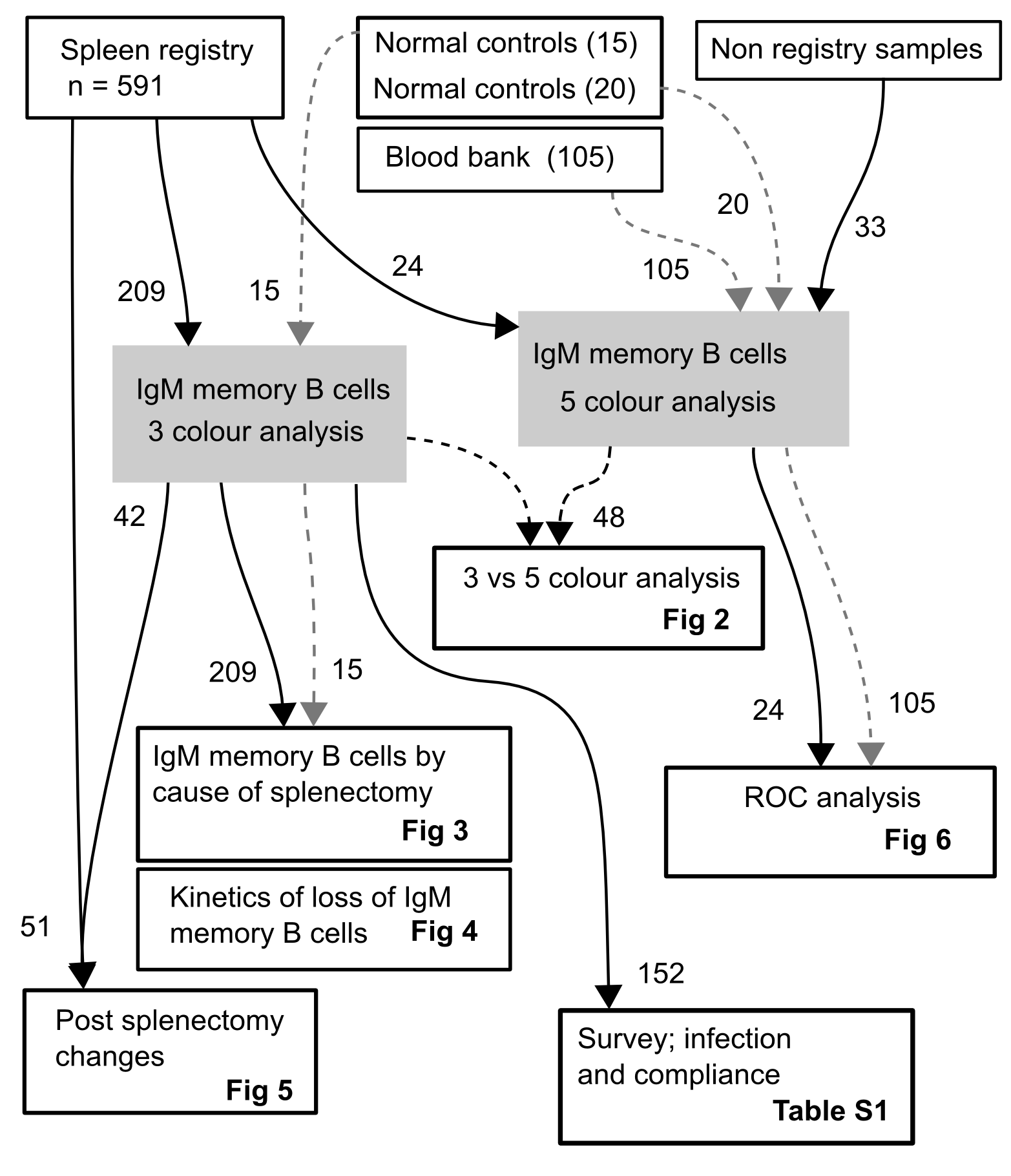

Supplement: Figure S1 — Patient samples used in the study. IgM memory B cell assays were performed at the time of entry for 209 of the the registry patients (n = 591). This populations was used to determine relationship between cause for splenectomy and IgM memory B cells and in the analysis of changes in IgM memory B cells with time following splenectomy. A subset of “incident” patients was used to determine haematological changes in comparison to IgM memory B cells changes. 51 patients had haematological data from the time of splenectomy of which 45 had an initial blood film that did not show HJB and follow-up blood films. Of these 42 had measurement of IgM memory B cells including 12 with sequential measures. Two assays for IgM memory B cells were used in this study; an initial 3 colour analysis that was compared to a normal control population (n = 15) and later a 5 colour assay that was compared to a normal control population (n = 20). These 2 assays were directly compared for 48 samples including some non-registry patient samples and registry patients. The reference values for this assay were established using a normal blood bank donor population that was selected to cover the adult age range and equally represent male and females. This was compared by ROC analysis against a splenectomy population that was more than 1 year post-splenectomy (n = 24). Data was obtained from patient survey of compliance with spleen registry recommendations. The subset of patients tested for IgM memory B cells (n = 152) was then used to determine relationship between the parameter “IgM memory B cells/B cells” and measures of compliance and indicators of infection. (TIF) [file pone.0023164.s001.tif]

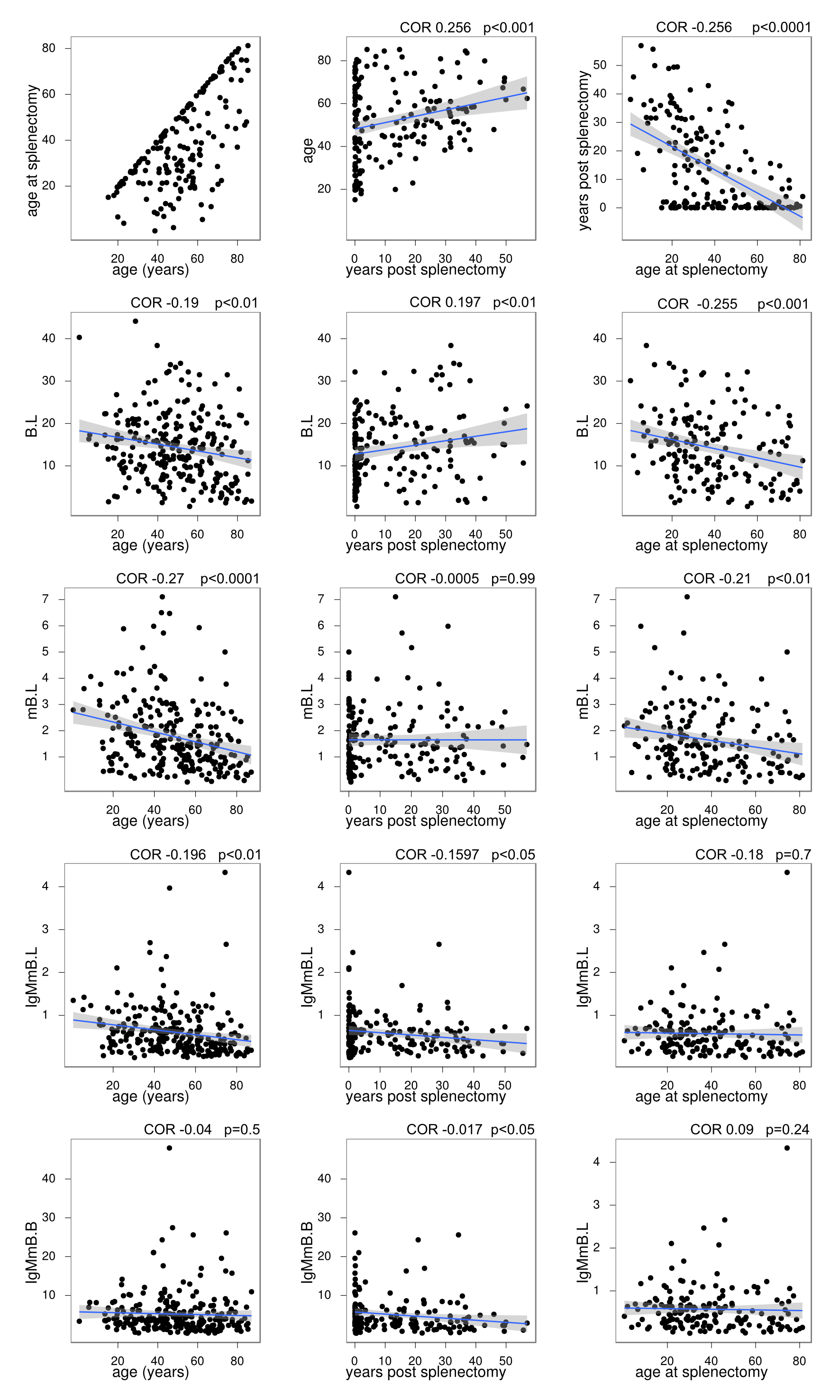

Supplement: Figure S2 — Correlation of changes in B cell subsets with age at splenectomy and time post splenectomy. Patients after splenectomy were analyzed for changes in proportion of B cells, memory B cells, IgM memory B cells with age (left column). The variable of changes in B cell with age was resolved into comparisons of changes in B cells with time since splenectomy (middle column) and changes with age at the time of splenectomy (right column). The Pearson correlation and p values for the correlations are shown above each plot. (TIF) [file pone.0023164.s002.tif]

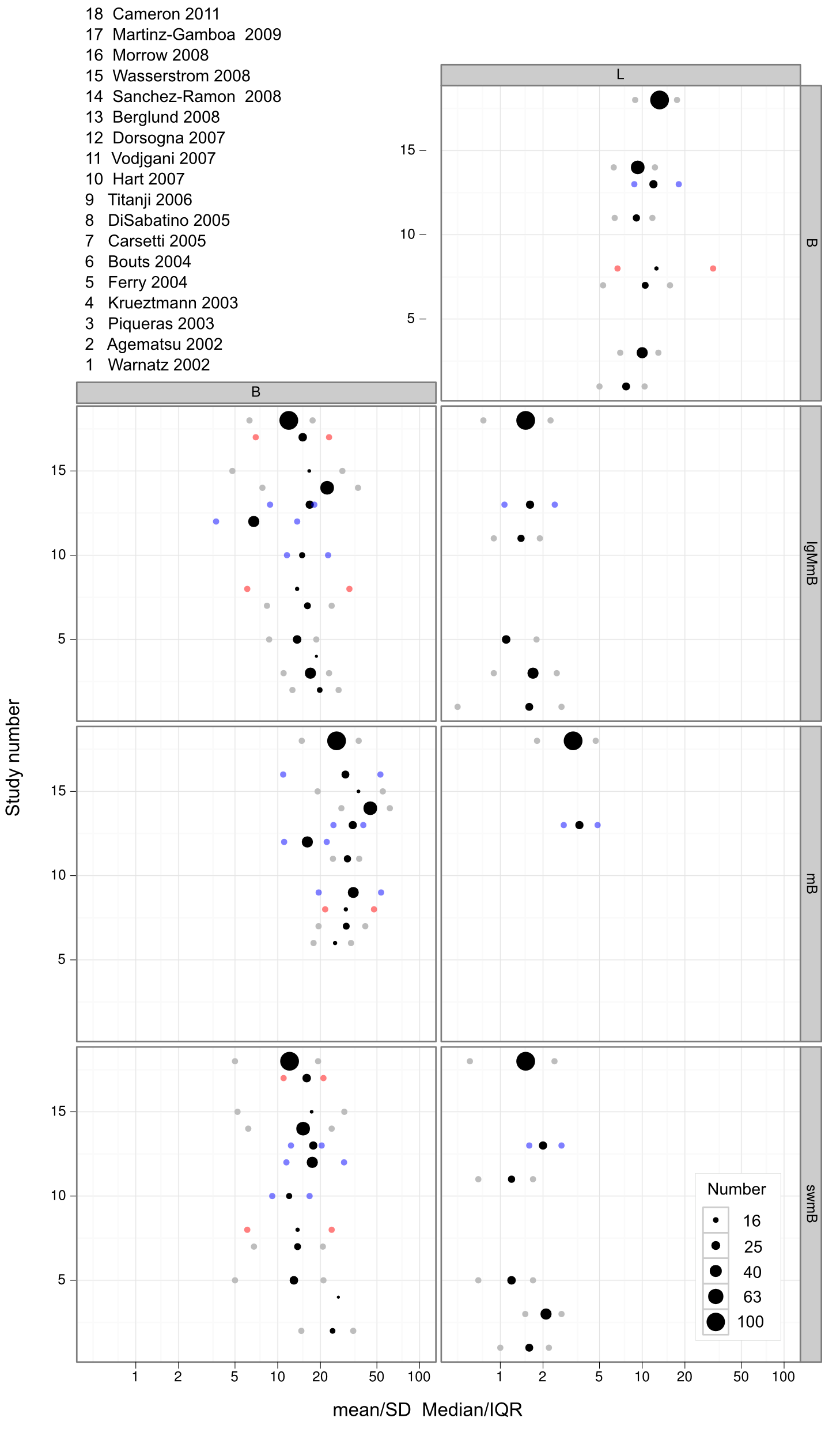

Supplement: Figure S3 — Comparison of reference range from the current study with other studies of IgM memory B cells and B cell subsets. The summary data for healthy control populations were taken from published studies and plotted as mean and SD, median and IQR (blue) or median and range (red). The size of the mean/median symbol is proportional to the log of the number of subjects in the reference population. Each cell type is shown as proportion of B cells (B) or of total lymphocytes (L). (TIF) [file pone.0023164.s003.tif]
