# Supplementary material for: Splenectomy Associated Changes in IgM Memory B Cells in an Adult Spleen Registry Cohort
Source: PLoS One. 2011 Aug 4;6(8):e23164. doi: 10.1371/journal.pone.0023164 (PMC3150402; doi:10.1371/journal.pone.0023164)
Supplement: Table S1 — IgM memory B cells as a predictor of infectious complications after splenectomy. Patients were divided into 2 groups based on IgM memory B cell levels above (>cutoff) or below (<cutoff) the cutoff determined by the ROC analsysis shown in fig. 6. The 2 groups where then compared for parameters determined from the patient survey. The initial analysis (upper panel) found significant association with absent HJB and had a nuber of patients at an early time post splenectomy. The analysis was repeated after exclusion of subjects where the IgM memory B cells had been determine less than 200 days after splenectomy. (DOC) [file pone.0023164.s004.doc]

Table S1

IgM memory B cells/B cells as a predictor of infectious complications after splenectomy

| **At enrolment** | **IgM memory B / B cells *** | |  |
| --- | --- | --- | --- |
|  | > cutoff (n=64) | < cutoff (n= 88) | p value# |
| Age median in years (range) | 47.62 (15.2-84.5) | 52.62 (18.0-84.5) | 0.46 |
| Time post-splenectomy years (range) | 2.08 (0.01- 46) | 9.32(0.01- 56.9) | 0.08 |
| Male | 32/63 (50.7%) | 45/88(51.1%) | 0.97 |
| HJB present | 25/44(56.8%) | 59/71(83%) | 0..002 |
| **More than 200 days after splenectomy** | | | |
|  | >cutoff (n=29) | <cutoff (n=59) |  |
| Age median in years (range) | 52.82 (32.7-84.5) | 55.65 (18.0-85.3) | 0.89 |
| Time post-splenectomy years (range) | 20.7 (0.3-45.7) | 13.2 (0.3-56.7 ) | 0.5 |
| Male | 9/28 (32.1%) | 28/59 (47.5%) | 0.18 |
| HJB present | 15/29 (52%) | 42/59 (71%) | 0.07 |
| Infection last 12 months | 10/29 (34.5%) | 18/59(30.5%) | 0.71 |
| Hospitalized with infections | 4/29 (13.8%) | 6/59 (10.2%) | 0.88 |
| Infection treated antibiotics | 4/29 (13.8%) | 9/59 (15.3%) | 0.89 |
| Prophylactic antibiotics | 9/29 (31%) | 26/59 (44.1%) | 0.24 |
| Emergency antibiotics | 16/29 ( 55.2%) | 35/59 (59.3%) | 0.71 |
| Any vaccine booster in 12 months | 13/29(44.8%) | 21/59 (35.6%) | 0.40 |
| Flu vaccine | 25/29(86.2%) | 49/59 (83.1%) | 0.94 |
| H1N1 vaccine | 15/29 (51.7%) | 34/59 (57.6%) | 0.60 |

* cutoff determined from figure 6 . # chisq test for categorical variable and wilcoxon test for continuous variables.
